# Formaldehyde-Assisted Isolation of Regulatory Elements (FAIRE) Analysis Uncovers Broad Changes in Chromatin Structure Resulting from Hexavalent Chromium Exposure

**DOI:** 10.1371/journal.pone.0097849

**Published:** 2014-05-16

**Authors:** Jerald L. Ovesen, Yunxia Fan, Xiang Zhang, Jing Chen, Mario Medvedovic, Ying Xia, Alvaro Puga

**Affiliations:** Department of Environmental Health and Center for Environmental Genetics, University of Cincinnati College of Medicine, Cincinnati, Ohio, United States of America; Florida State University, United States of America

## Abstract

The ability of chromatin to switch back and forth from open euchromatin to closed heterochromatin is vital for transcriptional regulation and genomic stability, but its dynamic structure is subject to disruption by exposure to environmental agents such as hexavalent chromium. Cr(VI) exposure disrupts chromatin remodeling mechanisms and causes chromosomal damage through formation of free radicals, Cr-DNA adducts, and DNA-Cr-protein cross-links. In addition, acute, high-concentration, and chronic, low-concentration exposures to Cr(VI) lead to significantly different transcriptional and genomic stability outcomes. We used mouse hepatoma Hepa-1c1c7 cells to investigate how transcriptional responses to chromium treatment might correlate with structural chromatin changes. We used Formaldehyde-Assisted Isolation of Regulatory Elements (FAIRE) analysis coupled with deep sequencing to identify regions of the genome that may switch between open and closed chromatin in response to exposure to varying Cr(VI) concentrations. At either Cr(VI) concentration, chromatin domains surrounding binding sites for AP-1 transcription factors become significantly open, whereas BACH2 and CTCF binding sites are open solely at the low and high concentrations, respectively. Parallel gene expression profiling using RNA-seq indicates that the structural chromatin changes caused by Cr(VI) affect gene expression levels in the target areas that vary depending on Cr(VI) concentration, but show no correlation between global changes in the overall transcriptional response and Cr(VI) concentration. Our results suggest that FAIRE may be a useful technique to map chromatin elements targeted by DNA damaging agents for which there is no prior knowledge of their specificity, and to identify subsequent transcriptomic changes induced by those agents.

## Introduction

The ability to control gene expression depends on critical epigenetic components that regulate chromatin structure [1]. Transcriptional complexes may cause changes in chromatin structure and chromatin structure in turn may serve as a key regulator of transcriptional activity. Heterochromatin, where DNA is tightly bound to histones, serves to block access to regulatory gene elements by transcription factors, while more loosely packed euchromatin allows the transcriptional machinery to have greater access to the DNA [2]. In order to regulate DNA access, transcription factors binding to their recognition motifs target the recruitment of many cofactors to alter chromatin structure and either loosen or tighten DNA-histone interactions [3]. These cofactors can either actively remodel chromatin structure, such as is the case of SWI/SNF complexes [4], or alter chromatin structure through posttranslational modifications on the tails of DNA associated histones, as histone acetyltransferases (HAT) and histone methyltransferases (HMT) do [5]–[7]. The binding of transcription factors and the recruitment of these co-factors leads to a cascade of events that alter chromatin structure, transitioning from heterochromatin to euchromatin and back to heterochromatin, as may be needed in response to stimuli that regulate transcription factor access to genes and the resulting gene expression [3].

The negative health effects of exposure to hexavalent chromium, especially lung cancer, have been recognized for more than 100 years [8], [9]. Cr(VI), used in many industrial processes and found in the waste of many different industries, is known to be a powerful carcinogen and mutagen [10], [11]. The basic foundations of the mechanism of Cr(VI) toxicity are fairly well understood. Sulfate ion transporters facilitate Cr(VI) transport into the cells; once inside, Cr(VI) is reduced through 1-electron intermediates Cr(V) and Cr(IV) to stable Cr(III), producing reactive oxygen species and causing radical-mediated DNA damage [12]. Cr(VI) reduction leads to the formation of stable Cr complexes with DNA and proteins causing formation of Cr-DNA adducts, DNA-Cr-DNA crosslinks, and DNA-Cr-protein crosslinks [13], although a recent study has shown that formation of DNA-Cr-DNA crosslinks is an *in vitro* phenomenon that does not occur in living cells [14]. These Cr adducts disrupt the mechanisms of chromatin remodeling leading to the alteration of normal gene expression processes [15]–[17]. Adducts and crosslinks seem to preferentially take place at sites of high DNA replication and transcription activity and often include crosslinking of chromatin remodeling complexes containing proteins of the epigenetic machinery [18], [19]. Cr-DNA adducts, and DNA-Cr-protein crosslinks, especially those containing chromatin remodeling complex components, cause disruption of chromatin structure throughout the nucleus, changing gene expression and potentially altering normal cellular growth patterns and responses to stress.

Formaldehyde-assisted isolation of regulatory elements (FAIRE) utilizes DNA/protein crosslinking followed by phenol/chloroform extraction to differentially segregate regions of the genome as determined by the amount of histone/DNA contacts that can be cross-linked [20], [21]. FAIRE can be used to identify and analyze active regulatory sequences based on their decreased nucleosomal content and, when combined with high-throughput sequencing (FAIRE-seq), to locate tissue-specific regulatory elements at a genome-wide scale [22], [23]. Given the disruption of chromatin structure caused by Cr treatment, we argued that FAIRE could be used to identify dynamic changes in regions of the genome open or closed by Cr exposure. To test this hypothesis, we compared FAIRE signals of untreated hepatoma cells with signals in cells treated with two extreme protocols of Cr(VI) exposure; one, an acute high concentration of 25 µM Cr(VI) for 90 minutes and the other, a sustained growth for 20 passages in the presence of a low concentration of 0.5 µM Cr(VI). We identified AP-1, BACH2 and CTCF/BORIS as specific Cr concentration-dependent FAIRE signatures in chromatin. When FAIRE-seq results were combined with RNA-seq data from the same cells, we found that the structural chromatin changes that occur as a response to Cr(VI) treatment do not seem to correlate with any changes in the overall transcriptional response caused by Cr(VI) treatment. These observations suggest that chromatin structural changes might contribute to, but by themselves are insufficient to cause the gene transcription changes induced by Cr. Nonetheless, FAIRE appears to be effective at mapping chromatin elements targeted by DNA damaging agents for which there is no prior knowledge of their specificity.

## Materials and Methods

### Cells and Treatments

Hepa-1c1c7 (Hepa-1) mouse hepatoma cells from the American Tissue Culture Collection were maintained in α-minimum essential media (α-MEM, Gibco) with 5%(v/v) fetal bovine serum (Sigma) and 1% (v/v) antibiotic-antimycotic (Gibco) in a 5% CO_2_ humidified atmosphere at 37°C. Cells were passaged at a 1∶6 ratio when they reached 80% confluence, typically every third day. Cells exposed acutely with high concentration Cr(VI) were treated with a final concentration of 25 µM CrK_2_O_4_ in their growth medium for 90 minutes. The medium of cells chronically exposed to low concentration Cr(VI) was supplemented with 0.5 µM CrK_2_O_4_ every 24 hours and the cells were grown for a total of 20 passages, approximately equivalent to 50 cell divisions. Control cells were passaged for the same number of passages in the absence of chromium.

### FAIRE and FAIRE-Seq

Cells were treated with 1% formaldehyde at room temperature for five minutes to form DNA-protein crosslinks and the crosslinking was stopped by addition of glycine to a final concentration of 125 mM. Cells were pelleted, washed 3 times in 4°C PBS and lysed in cell lysis buffer (10 mM Tris-HCl, 10 mM NaCl, 3 mM MgCl_2_, 0.5% NP-40, and proteinase inhibitors) on ice for 10 minutes. Nuclei were pelleted and lysed in nuclei lysis buffer (10 mM EDTA, 50 mM NaCl, 1% SDS and proteinase inhibitors) on ice for 10 minutes. Lysates were sonicated in a sonic Bioruptor (Diagenode) and diluted with 50% v/v dilution buffer (12 mM EDTA, 17 mM Tris-HCl, 167 mM NaCl, 0.01% Triton X-100, 0.01% SDS). Cell debris were removed by micro-centrifugation and free DNA was extracted from the collected supernatant by phenol/chloroform extraction. Under these conditions, DNA not crosslinked to proteins remains in the aqueous phase while the DNA crosslinked to proteins remains in the phenol phase [20]. For FAIRE-seq, purified free DNA fragments of 200±100 bp in size were sequenced with Illumina HiSeq system. In brief, the indexed sequencing library was constructed using Ovation Ultralow Library System (NuGEN) according to the manufacturer protocol. The library was analyzed for quality control by 2100 Bioanalyzer (Agilent) and real-time PCR quantified using a Library Quantification kit (Kapa Biosystems). The equal-amount pooled libraries were clustered onto a flow cell using the TruSeq SR Cluster kit v3 (Illumina) in an Illumina cBot system. The pooled libraries were sequenced using the TrueSeq SBS v3 kit for 50 cycles for approximately 30 million reads per sample, which were aligned to the mouse genome using Illumina’s standard sequence alignment pipeline.

### RNA Isolation and RNA-Seq

Total RNA was isolated using the Qiagen’s RNeasy kit per the manufacturer protocol. Quality control analysis of the RNA was performed using the Agilent 2100 Bioanalyzer. Using TruSeq RNA sample preparation kit (Illumina), poly-A mRNA was extracted from the total RNA with RNA integrity number (RIN, Agilent 2100 Bioanalyzer) ≥7.0, followed by RNA fragmentation and double strand cDNA conversion. The sequencing library was constructed according to the protocol followed by the library QC analysis, quantification, clustering and sequenced as described above. For each sample, approximately 30 million reads were aligned to the genome using Illumina’s standard sequence alignment pipeline.

### Data Analysis

RNA-seq data was analyzed as documented earlier [24]. Briefly, sequence reads were aligned to the reference mouse genome (build mm10) using TopHat. Differential expression analysis between treated and control cells was performed using the DESeq package in Bioconductor.

For FAIRE-seq data analysis, sequence reads from FAIRE experiments were first aligned to the same reference mouse genome as was used for RNA-seq data analysis using the BWA tool [25]. Thereafter, MACS (Model-based Analysis of ChIP-Seq) [26] was used to identify the peaks in Cr exposed samples relative to the corresponding control. Aligned BAM files from FAIRE-Seq data with default parameter settings were used in MACS analysis. The peaks detected by MACS were further annotated by the HOMER software [27]. Given the genomic locations of the peak regions, HOMER tested whether a particular genomic location (intron, exon, promoter, 5′UTR, 3′UTR, etc.) or a known transcription factor motif was enriched in the peaks and whether the enrichment was statistically significant. Genome-wide FAIRE-seq and RNA.seq data have been submitted to the GEO database with access URL http://www.ncbi.nlm.nih.gov/geo/query/acc.cgi?acc=GSE56636.

## Results

### Acute High Concentration and Long-term Low Concentration Chromium Treatment cause Very Different Changes to Chromatin Organization

**Figure 1:**
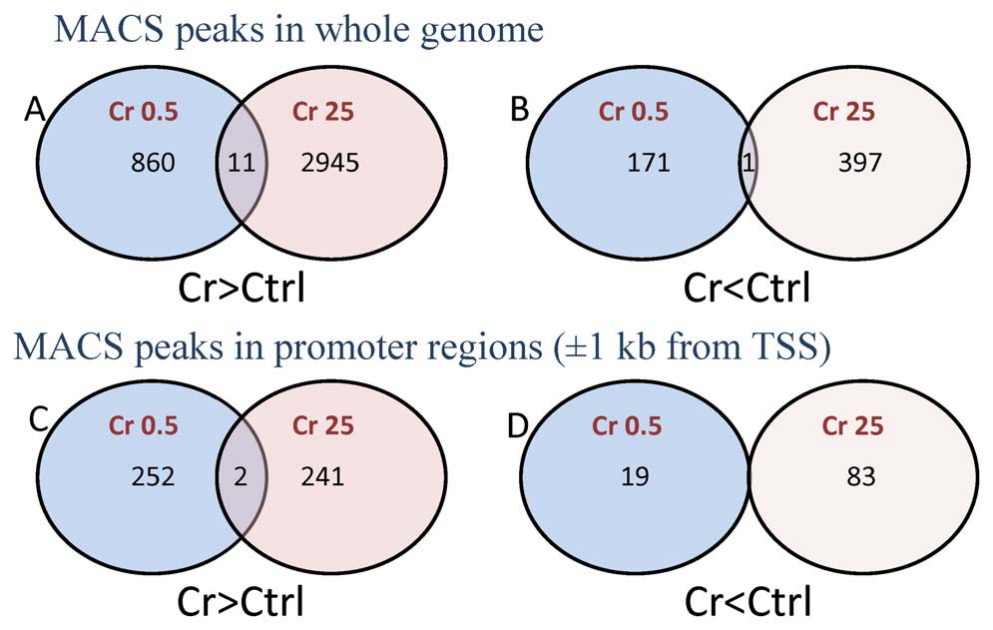
Long-term low dose and acute high dose Cr(VI) treatment affect chromatin structure very differently. *Top row:* Venn diagrams of FAIRE DNA peaks induced in Hepa-1 cells treated with 25 µM Cr(VI) for 90 minutes compared to peaks induced in cells grown in medium containing 0.5 µM Cr(VI) for 20 passages. The diagrams show the number of peaks (**A**), opened in Cr-treated cells relative to control, and (**B**), opened in control relative to Cr-treated cells. *Bottom row:* Venn diagrams corresponding to those in the *Top row* for MACS peaks in promoter regions as defined by a distance of ±1 kb from the TSS. The diagrams show the number of peaks (**C**), opened in Cr-treated cells relative to control, and (**D**), opened in control relative to Cr-treated cells.

FAIRE DNA induced in Hepa-1 cells by treatment with 25 µM Cr(VI) for 90 minutes showed 2956 MACS peaks corresponding to open chromatin regions, as defined by partitioning to the aqueous phase of the phenol-chloroform extraction and hence, not being associated with histones. In untreated control cells, these regions were closed, that is, they partitioned to the non-polar phase of the extraction protocol and hence, were histone associated (Fig. 1A). Conversely, 398 MACS peaks were open in control cells and closed in cells treated with 25 µM Cr(VI) (Fig. 1B). Of the 2956 regions that were open in acute Cr(VI) treated cells but not in control, only 243 were located within 1 kilobase-pair of a gene transcriptional start site (TSS) suggesting that less than 10% of the Cr(VI) induced chromatin openings might directly affect gene expression regulation from promoter regions (Fig. 1C). Similarly, only 83 of the 398 chromatin regions that were open in control cells and closed in cells treated by an acute 25 µM Cr(VI) concentration were within 1 kilobase-pair of a gene TSS (Fig. 1D).

Cells grown in medium containing 0.5 µM Cr(VI) for 20 passages were strikingly different from cells acutely treated with 25 µM Cr(VI) for 90 minutes. Continued treatment with low levels of Cr(VI) opened 871 and closed 172 chromatin regions relative to control cells (Fig. 1B). Of the 871 open chromatin peaks only 11 were found in common with the nearly 3,000 regions that were open in the acute high concentration Cr(VI) treatment (Fig. 1A). Similarly, of the peaks more open in control than in Cr-treated cells, only 1 of the 172 peaks open in the control cells passaged for 20 generations was found in common with the 398 peaks open in the control of the acutely treated cells.

When comparing peaks in regions of potential promoters affected by the two treatments, the differences were equally apparent. Approximately, the same number of open chromatin peaks were found in the acute, high concentration Cr(VI) treated cells as in the low concentration, long term passaged cells; 254 versus 243 peaks, respectively were located within 1 kb of potential TSSs, but only 2 sites were found in common in the two groups (Fig. 1C). Similarly, of the 83 peaks in potential promoter regions that were more closed in response to acute high concentration Cr(VI) exposure, none were found in the corresponding 19 peaks in control passaged cells that were within 1 kb of TSSs (Fig. 1D). Overall, the results from these analyses indicate that, although both Cr(VI) treatments tested cause concentration-specific structural changes in chromatin, these changes are not consistent between the two treatment regimens.

### Chromium-dependent Opening of FAIRE Peaks in Promoter Regions are Significantly Correlated with Gene Expression

**Figure 2:**
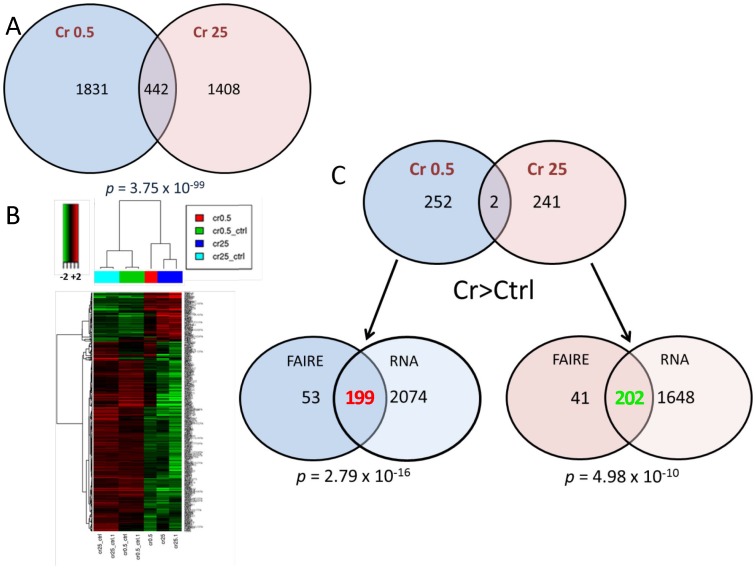
Opening of FAIRE peaks in promoter regions by chromium treatment is significantly correlated with changes in gene expression. (**A**) Venn diagram of concordant (*p* = 3.75×10^−99^) gene expression changes induced by long-term low dose and acute high dose Cr(VI) treatments. (**B**) Hierarchical clustering of the 442 genes with concordant expression. (**C**) gene expression changes corresponding to peaks opened by Cr(VI) in promoter regions within 1 kb of the TSS. In the low concentration, long term Cr(VI) treatment, 199 of the 252 FAIRE peaks were contained in up-regulated genes (*p* = 2.79×10^−16^) and 202 of 243 peaks in the acute, high concentration treatment were contained in down-regulated genes (*p* = 4.98×10^−10^).

FAIRE peaks identify DNA segments that are depleted of nucleosomes and likely to contain transcription regulatory elements [21]. To determine whether FAIRE could be used to characterize segments of the genome transcriptionally open by Cr(VI) treatment, we used RNA-seq analysis after either acute, high concentration or long-term low concentration Cr(VI) treatments. Acute exposure to 25 µM Cr(VI) altered the expression of 1850 genes, while long-term low concentration Cr(VI) treatment caused significant differential expression of 2273 genes. These two sets of genes were significantly concordant, with 442 genes affected by both treatments (Fisher exact test *p* = 3.75×10^−99^) (Fig. 2A). Hierarchical clustering of these genes showed a high degree of agreement between the two types of treatment in the direction–repression or induction–of the changes resulting from exposure (Fig. 2B). Similarly, gene expression changes corresponding to peaks opened by Cr(VI) in promoter regions within 1 kb of the TSS showed a striking degree of agreement with the type of Cr(VI) treatment. Of the 252 FAIRE peaks found in the low concentration, long term Cr(VI) treatment, 199 were contained in genes that were up-regulated (Fisher exact test *p* = 2.79×10^−16^); in contrast, of the 243 peaks found in the acute, high concentration treatment, 202 were contained in down-regulated genes (Fisher exact test *p* = 4.98×10^−10^). Remarkably, it appears that low and high concentration treatments cause opposite effects not only on the regions of the genome affected by treatment, but also in the direction of the resulting gene expression changes induced.

### Cr(VI) Exposure Preferentially Opens Chromatin in Promoters Containing CTCF and AP1 Motifs

**Figure 3:**
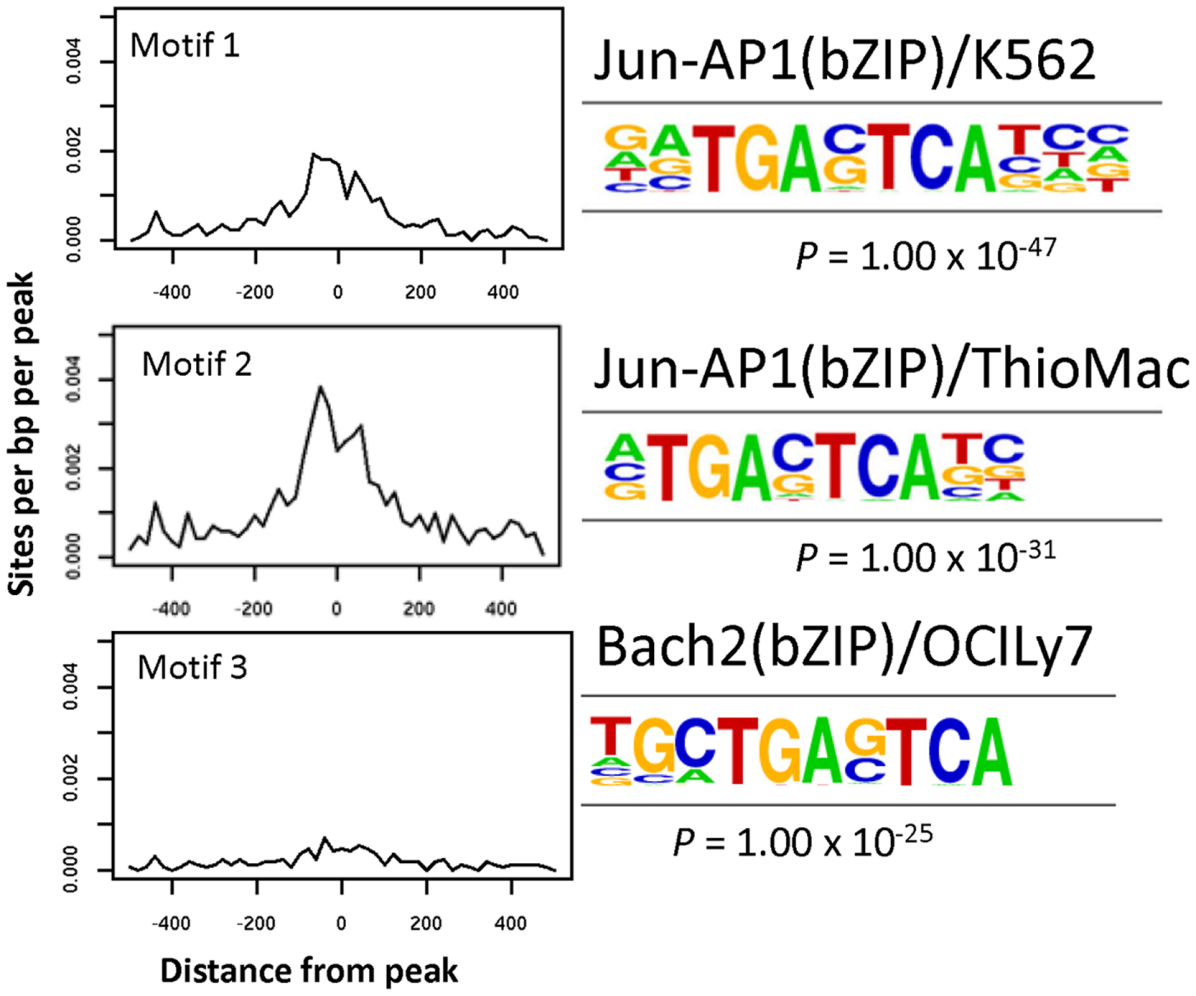
Long-term low concentration Cr(VI) treatment preferentially opens chromatin in promoters containing AP1 binding sites. HOMER analyses identified AP1 transcription factor binding motifs located within promoter elements significantly more often located in open chromatin of cells treated with a sustained low-concentration Cr(VI). The graphs on the left represent the number of times per base-pair per peak the indicated motifs are present within ±500 bp of the TSS. The logos on the right correspond to the position weight matrix identified by HOMER. Also shown is the Fisher exact test *p* value of the correlation.

**Figure 4:**
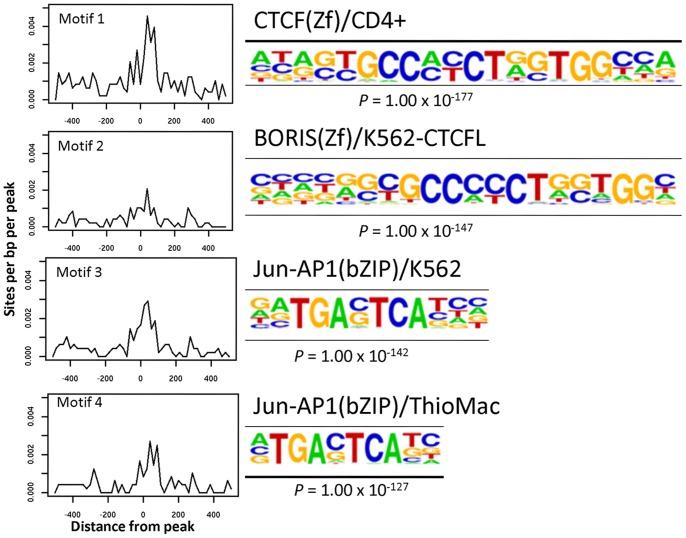
Acute, high concentration Cr(VI) treatment preferentially opens chromatin in promoters containing CTCF/BORIS and AP1 binding sites. HOMER analyses of peaks opened by acute, high concentration Cr(VI) treatment. Legend as in Figure 3.

HOMER analysis informs on different types of genomic elements appearing in the chromatin structures accessed by FAIRE. In both experimental Cr(VI) treatments, promoter elements, defined by HOMER as areas within 500 base pairs of a TSS, were significantly enriched in the open chromatin, to nearly 16 times relative to random chance in the low concentration long-term treated cells and almost 4 times in the acutely treated cells (Table 1). HOMER analyses also identified transcription factor binding motifs located within open chromatin of cells treated with high- or low-concentration that significantly more often than random regions of same lengths in similar promoters. The low-concentration chronic treatment caused opening of chromatin enriched for two different position weight matrices of the binding motif for the transcription factor AP-1 (*p = *1×10^−47^), as well as a motif recognized by BACH2 (*p* = 1×10^−25^), a transcription factor involved in immune system tolerance [28] (Fig. 3). The acute, high concentration Cr(VI) treatment opened binding motifs for the transcriptional repressor CTCF (*p* = 1×10^−177^), the CTCF paralog BORIS (*p* = 1×10^−147^), as well as the same two binding motifs for the AP-1 transcriptional complex (*p* = 1×10^−142^ and 1×10^−127^, respectively) observed in the low concentration treatment (Fig. 4). The AP-1 binding motif is present in 15 out of the 135 predicted promoter sites found in chromatin open in response to long term exposure to 0.5 µM Cr(VI) and in 10 out of 106 promoter sites in chromatin opened by acute exposure to 25 µM Cr(VI) (Tables 1 and 2). None of these genes were shown by RNA-seq to have significantly changed gene expression levels in response to Cr(VI) treatment. These results suggest that even though Cr(VI) may induce broad changes in the structure of chromatin, these changes are not necessarily followed by concomitant alterations in gene expression. Furthermore, the preference for transcription factor binding sites demonstrated by Cr(VI) under both extreme treatment regimens is limited to the primary sequence of the chromatin domain and, judging from the genes affected, does not show evidence of gene specificity.

**Table 1:** Enrichment of Genomic Elements in FAIRE Peaks Opened by Cr(VI) Treatment.

	Long-term Low Cr(VI) Concentration		Acute, High Cr(VI) Concentration	
Annotation	Number of Peaks	Fold Enrichment	Number of Peaks	Fold Enrichment
3UTR	11	1.82	23	1.11
5UTR	3	4.77	2	0.93
Exon	5	0.48	28	0.79
Intergenic	436	0.81	1441	0.78
Intron	260	0.90	1303	1.32
miRNA	0	0.00	0	0.00
miscRNA	0	0.00	7	2.57
Promoter	135	15.93	106	3.65
pseudo	0	0.00	0	0.00
rRNA	1	561.83	0	0.00
snoRNA	0	0.00	0	0.00
TTS	9	1.13	35	1.28

**Table 2:** Genes with AP1-Containing Promoters Opened by Cr(VI) Treatment.

A. Genes with AP1-Containing Promoters opened by 0.5 µM Cr(VI)	
**Gene Name**	**Gene Description**
Ccdc50	Coiled-coil domain containing 50
Cdc42se2	CDC42 small effector 2
Cirbp	Cold inducible RNA binding protein
Ctsa	Cathepsin A
Fnbp1	Formin binding protein 1
Gm16853	Predicted gene, 16853
Gm3448	Predicted gene 3448
Lrrfip1	Leucine rich repeat (in FLII) interacting protein 1
Mmp10	Matrix metallopeptidase 10
Mrpl4	Mitochondrial ribosomal protein L4
Pcmtd1	Protein-L-isoaspartate (D-aspartate) O-methyltransferase domain containing 1
Psmd12	Proteasome (prosome, macropain) 26S subunit, non-ATPase, 12
Rtel1	Regulator of telomere elongation helicase 1
Slc3a1	Solute carrier family 3, member 1
Trappc10	Trafficking protein particle complex 10
**B. Genes with AP1-Containing Promoters opened by 25 µM Cr(VI)**	
1700022P22Rik	RIKEN cDNA 1700022P22 gene
Gcnt2	Glucosaminyl (N-acetyl) transferase 2, I-branching enzyme
Gjc2	Gap junction protein, gamma 2
Gm19461	Predicted gene, 19461
Mir3097	MicroRNA 3097
Ndufs7	NADH dehydrogenase (ubiquinone) Fe-S protein 7
Pgap3	Post-GPI attachment to proteins 3
Slc29a1	Solute carrier family 29 (nucleoside transporters), member 1
Slc4a11	Solute carrier family 4, sodium bicarbonate transporter-like, member 11
Wbp1l	WW domain binding protein 1 like

## Discussion

The results that we describe here show that Cr(VI) exposure causes large and diverse structural changes in chromatin conformation. After acute treatment with 25 µM Cr(VI), nearly three times (2956/871) as many unique chromatin domains are opened than after sustained exposure to 0.5 µM Cr(VI) for 20 cell passages, yet only 11 of these domains are shared between the two treatments, suggesting that different treatment levels may trigger different mechanisms of action and ultimately show markedly different biological outcomes. We have previously shown that Cr(VI) can cross-link chromatin remodeling complexes to specific DNA promoters [19] and that Cr(VI) exposure causes DNA damage that alters cellular gene expression and the response to other external stimuli, such as benzo[*a*]pyrene exposure [29]. This observation is confirmed by our current results, to the extent that different treatment concentrations cause different gene expression responses.

We have examined what if any changes Cr(VI) causes to the overall dynamics of cellular chromatin. Interestingly, changes in specific chromatin domains under one treatment condition do not seem to be predictive of similar changes in that same chromatin domain under the other treatment. It is likely that the differences that we observe between chronic and acute treatments are due to selective pressures for cell survival in the long-term chronic treatment. Our previous data showed that long-term Cr(VI) treatment led to DNA damage and increased expression of apoptosis markers, even though the cells adapted and continued to proliferate, albeit changing gene expression patterns [29]. On the other hand, the 25 µM Cr(VI) concentration used for the acute treatment would be a lethal dose for Hepa-1 cells if extended much beyond the short treatment time; hence the changes detected at this concentration are likely to be terminal. Regardless of the state, open or closed, of the chromatin, all genes with chromatin domains located in promoters altered by acute Cr(VI) treatment were down-regulated compared to control, and the opposite was true for cells chronically grown in the presence of 0.5 µM Cr(VI) for 20 passages, which were all up-regulated, regardless of their location in more closed or more open chromatin regions. This observation further suggests that the cellular mechanisms that each treatment disrupts and the cellular responses that each treatment elicits are different and distinct. An interesting possibility is that acute high-concentration and chronic low concentration treatments elicit different DNA repair mechanisms, with specific repair pathways becoming constitutively active in surviving chronically treated cells. The distinction would make it difficult to predict the expected results of low-concentration treatment by extrapolating from data on high-concentration treatment, and *vice versa*.

Our work also exemplifies the random nature of the structural changes that chromium treatment causes in chromatin. Chromatin domains that are more open in either of our treatments are greatly enriched for promoter motifs, yet there are very few conserved transcription factor binding motifs that are statistically more prevalent in the open motifs of Cr(VI) than in control cells. The most likely explanation for this observation is that Cr(VI) effects on chromatin structure are not targeted, but are the result of random interactions with DNA and chromatin remodeling proteins. In this context, it is worth noting that Cr(VI) causes extensive formation of Cr-DNA adducts that are removed by the nucleotide excision repair system [30], it is possible that the randomness of chromatin changes detected by FAIRE is but a reflection of the lack of sequence-specificity in Cr-DNA adduction and nucleotide disruption and chromatin remodeling associated with tnucleotide excision repair. We have shown that acute Cr(VI) treatment cross-links proteins responsible for chromatin remodeling process to the DNA domains where they are already bound [19]. In all likelihood, this process occurs at random wherever those proteins are located, serving to lock them into place and forcing a resetting of chromatin structure at that location. In cells undergoing an acute treatment, these open areas may simply mark the position of cellular processes ongoing at the time of treatment and not be specific for any particular cellular response. In cells subjected to a sustained chronic treatment, the areas that remain open would be those whose chromatin structure does not disrupt cellular viability, which may or may not reflect any single cellular response. For both treatments, we find that the AP1 transcription factor binding motif appears to be opened more often in response to Cr(VI) than in control cells, either as a result of a chemical preference of Cr(VI) to interact with the TGANTCA motif, or of the selective nature of the treatment. In addition, acute treatment also opens up the CTCF/BORIS binding motif, generally associated with gene repression [31], [32]. Perhaps the regulation of the expression of genes bearing these motifs is less dependent on chromatin structure than on the regulation of the corresponding transcription factors. In fact, our data shows that the heterochromatin/euchromatin changes that occur as a response to Cr(VI) treatment do not seem to correlate with any changes in the overall transcriptional response caused by Cr(VI) treatment. Whether associated with euchromatin or heterochromatin, the expression of genes that were significantly altered by either Cr(VI) treatment was changed in the same direction. Possibly, Cr opens up chromatin in a dose independent fashion, but the dose determines whether the outcome is transcriptional activation or repression. In this regard, it is significant that the AP1 site is more frequent in the 199 genes common to FAIRE-seq and RNA-seq in cells grown in medium with 0.5 µM Cr(VI), which are induced by treatment, whereas the repressive CTCF site is more frequent in the 202 genes common to FAIRE-seq and RNA-seq in cells acutely treated with 25 µM Cr(VI), which are repressed.

There are multiple mechanism by which Cr(VI) can cause damage to cells and disrupt genomic structure and gene transcription. Cr(VI) can cause free radical induced mutations, crosslink transcription factors to DNA and block further activation of the associated promoters, disrupt chromosome structure by creating DNA-DNA crosslinks, and can itself serve to create a bulky adduct in the DNA [15]–[17], [33]–[35]. These adducts occur at locations of high transcription and replication activity, most likely due to the accessible nature of the chromatin in these locations. Our data however, show that beyond accessibility, Cr(VI) seems not to target with high frequency any particular DNA motif or set of genes for specific remodeling; rather, it appears more likely that it targets global chromatin topography. Furthermore, for our single high concentration acute and low concentration chronic treatments, the cellular response and the mechanism of action vary greatly, making extrapolation from one type of exposure to predict how a different exposure will affect cells, or an organism, extremely uncertain.
